# Genetic Polymorphisms Related to Testosterone Metabolism in Intellectually Gifted Boys

**DOI:** 10.1371/journal.pone.0054751

**Published:** 2013-01-30

**Authors:** Peter Celec, Denisa Tretinárová, Gabriel Minárik, Andrej Ficek, Tomáš Szemes, Silvia Lakatošová, Eva Schmidtová, Ján Turňa, Ľudevít Kádaši, Daniela Ostatníková

**Affiliations:** 1 Institute of Molecular Biomedicine, Comenius University, Bratislava, Slovak Republic; 2 Institute of Pathophysiology, Comenius University, Bratislava, Slovak Republic; 3 Department of Molecular Biology, Comenius University, Bratislava, Slovak Republic; 4 Institute of Molecular Physiology and Genetics, Slovak Academy of Sciences, Bratislava, Slovak Republic; 5 Institute of Physiology, Comenius University, Bratislava, Slovak Republic; Thomas Jefferson University, United States of America

## Abstract

Prepubertal testosterone levels are lower in intellectually gifted boys. The aim of this pilot study was to analyze potential genetic factors related to testosterone metabolism in control and gifted boys. Intellectually gifted (IQ>130; n = 95) and control (n = 67) boys were genotyped. Polymorphisms of interests were chosen in genes including androgen and estrogen receptors, 5-alpha reductase, aromatase and sex hormone binding globulin. Significant differences between control and gifted boys in genotype distributions were found for ESR2 (rs928554) and SHBG (rs1799941). A significantly lower number of CAG repeats in the AR gene were found in gifted boys. Our results support the role of genetic factors related to testosterone metabolism in intellectual giftedness. Increased androgen signaling might explain previous results of lower testosterone levels in intellectually gifted boys and add to the understanding of variability in cognitive abilities.

## Introduction

General intelligence is a highly heritable trait. Numerous epidemiological studies have shown that intelligence has a strong genetic influence that even increases with age [1], [2]. The heritability is estimated to be between 60% and 80% [3]. A strong gene-environment interaction is suggested based on epidemiological data [4]. Despite intense effort to identify the underlying genetic factors, there is still no major breakthrough in sight. One of the possible reasons is that the heritability is due to a large number of genes with very small individual informative value.

Genome-wide association studies are a strong research tool to find polymorphisms associated with diseases or physiological phenotypes. They proved to be less successful in finding genetic factors influencing intelligence. The numerous studies differed in the resolution of genome analysis, number of subjects and other methodological parameters [5], [6], [7], [8], [9], [10]. The studies, however, revealed no major association and more importantly, the results are not reproducible. These facts indicate that candidate gene analysis might be more suitable in this trait.

Gender differences in cognition are well-known [11] and usually attributed to endocrine differences between sexes. In a large meta-analysis it was shown that there is a slight but consistent gender bias in intelligence in favor of men [12]. In the top 3% of intellectually gifted children there are 12-times more boys than girls [13]. The main endocrine difference between sexes is the different concentration of sex hormones with especially high testosterone levels in men. Testosterone and its effects on cognitive abilities have been studied for many years [14]. The association between testosterone and general intelligence is, however, not clear. Epidemiological studies have brought indices for an association between testosterone and intelligence in all age groups [15], [16], [17], [18]. Our own research on prepubertal boys indicated that the levels of salivary testosterone are lower in intellectually gifted than in the control prepubertal population [19].

There are numerous genes related to testosterone metabolism and, thus, might represent candidate genes for this study. However, based on previous research analyzing genetic polymorphisms in relation to other cognitive traits we have chosen genes encoding proteins related to the action of testosterone [20]. A similar but larger study was conducted to analyze the association of genetic polymorphisms related to sex hormone metabolism and autism [21]. Sex hormone binding globulin (SHBG) binds testosterone in plasma and determines the free bioactive fraction of testosterone. Aromatase and 5-alpha reductase convert testosterone into estradiol and dihydrotestosterone, respectively. Polymorphisms of the corresponding genes were studied in relation to psychological traits [20], [22]. By binding to the androgen receptor (testosterone and dihydrotestosterone) or the estrogen receptor (estradiol) the effect of testosterone is mediated to the cells. Variants of these genes modulate the risk for some psychiatric diseases [23], [24].

The aim of our pilot study was to analyze the frequency of variants of selected polymorphisms of genes related to testosterone metabolism in intellectually gifted and control boys. We hypothesized that these polymorphisms will be associated with intellectual giftedness and will partially explain the association with testosterone levels found in a previous study.

## Materials and Methods

### Ethics Statement

The study was approved by a local ethic committee of the Faculty of Medicine, Comenius University in Bratislava. One of the parents signed an informed consent on behalf of every participating child. Written informed consent was obtained from the parents of all children involved in this study.

Boys were recruited from local schools in Bratislava self-governing region using presentations at the meeting of parents. Children willing to participate after agreement from the parents were asked to come to an informational meeting and for samples collection. Buccal swab samples were taken from 67 control boys (standard primary school; aged 14,8±1,2 years, IQ = 112,5±14,8) and 95 intellectually gifted boys (school for gifted children; aged 15,0±1,7 years, IQ = 143,2±9,6). The boys were all white Caucasians from the same geographical region and came from families with a similar socioeconomic status. IQ of 130 in the Stanford-Binet IQ test – fourth edition was the cut off for intellectual giftedness. Psychological testing and sampling was done by qualified personnel during one week in autumn. All samples were anonymized and coded.

DNA isolated from buccal swabs using a commercially available kit (QIAmp DNA Mini kit, Qiagen, Hilden, Germany) was used as the template for SNaPShot analysis (Applied Biosystems, Foster City, USA). Using primer extension “mini-sequencing” reactions with dideoxynucleotides and carefully designed oligonucleotides it is possible to genotype several single nucleotide polymorphisms (SNP) by capillary electrophoresis. The polymorphisms were chosen from the NCBI dbSNP database based on high heterozygosity in Caucasian population that assured the presence of various genotypes in such a small study group. All SNPs analyzed using the SNaPShot technology are presented in the manuscript. If problems with the readout occurred, standard Sanger sequencing was used for genotyping. In addition, the number of CAG repeats in the exon 1 of the androgen receptor gene was analyzed using PCR and capillary electrophoresis. Genes and all analyzed polymorphisms are summarized in Table 1.

**Table 1:** Polymorphisms of genes related to testosterone metabolism analyzed in this study.

SNP/STR	Gene	Description	Locus	Alleles
**rs700518**	CYP19A1	cytochrome P450 19A1; aromatase	15q21	A/G
**C1558T**	CYP19A1	cytochrome P450 19A1; aromatase	15q21	C/T
**rs2077647**	ESR1	estrogen receptor alpha	6q24-q27	A/G
**rs928554**	ESR2	estrogen receptor beta	14q21-q22	A/G
**rs1799941**	SHBG	sex hormone binding globulin precursor	17pter-p12	A/G
**rs9282858**	SRD5A2	3-oxo-5-alpha-steroid 4-dehydrogenase 2	2p23.1	C/T
**CAG STR**	AR	androgen receptor	Xq11-12	(CAG)n

XLGenetics and XLStatistics were used for the calculations and statistical analyses with Chi squared test. Bonferroni corrected p-values (nominally significant p-values were multiplied by the number of comparisons) less than the level of significance 0,05 were considered significant and are reported. The correction was applied to prevent multiple comparison bias as genotype frequencies were compared between the groups for several polymorphisms. Data are presented as genotype frequencies or means of tandem repeats with standard deviations.

## Results

**Figure 1:**
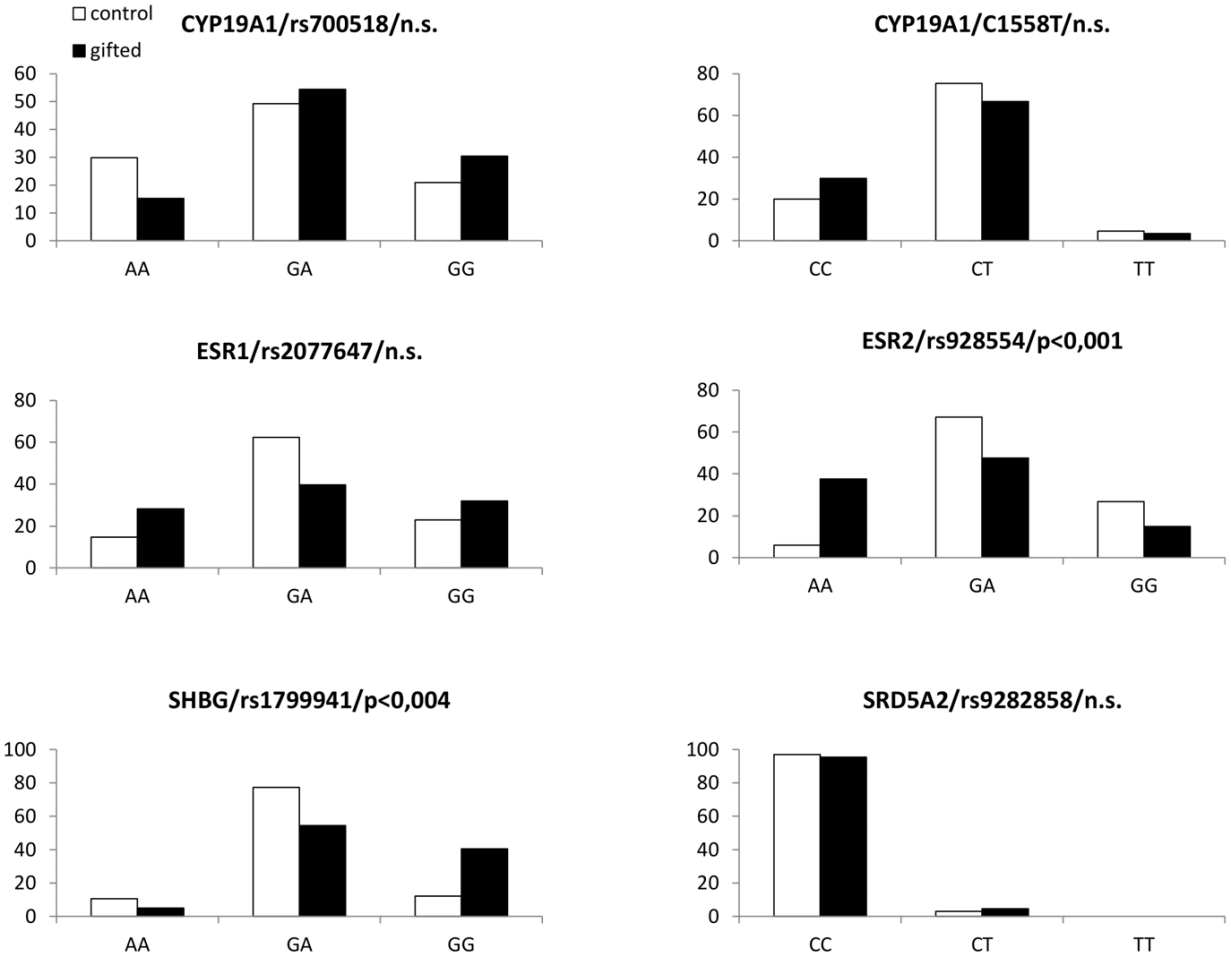
Genotype frequencies. Genotype frequencies (%) of the analyzed single nucleotide polymorphisms in intellectually gifted (black boxes) and control (white boxes) boys. Genotypes of the polymorphisms are shown below the graphs.

**Figure 2:**
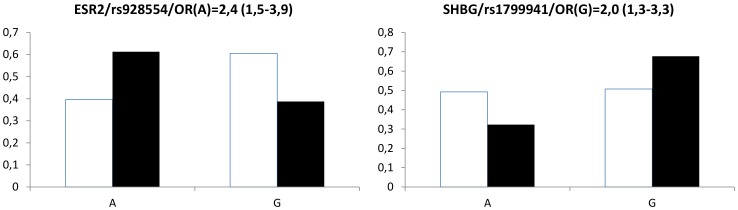
Allele frequencies. Allele frequencies of the single nucleotide polymorphisms that were significantly different distributed in intellectually gifted (black boxes) and control (white boxes) boys. Genotypes of the polymorphisms are shown below the graphs.

**Figure 3:**
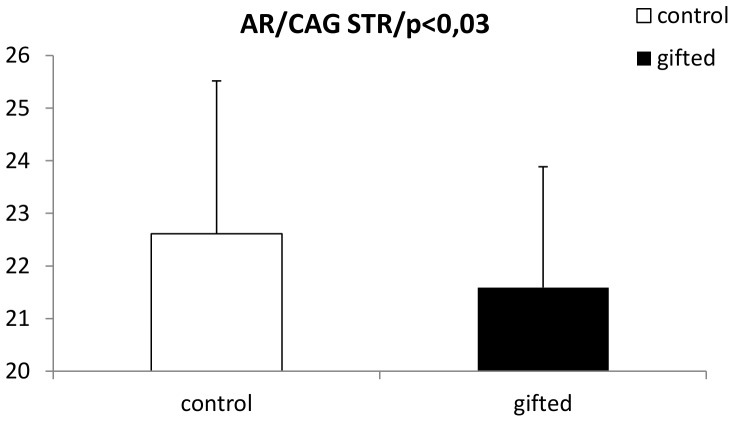
Androgen receptor gene. Number of CAG repeats in the first exon of the androgen receptor gene in intellectually gifted (black boxes) and control (white boxes) boys (data are presented as mean+standard deviation).

Genotype frequencies of the analyzed SNPs in control and intellectually gifted children are summarized on Fig. 1. All analyzed SNPs were in Hardy-Weinberg equilibrium. Significant differences in genotype distributions have been detected in ESR2 (rs928554; AA genotype is 8 times more common in gifted boys; χ^2^ = 20,7; p<0,001) and SHBG (r s179994; homozygotes are 3 times more common among gifted boys; χ^2^ = 14,9; p<0,004). The corresponding allele frequencies in these two polymorphisms are calculated and shown on Fig. 2. The short tandem repeat in exon 1 of the androgen receptor gene widely studied in association with prostate cancer is slightly but significantly shorter in intellectually gifted boys. The difference is approximately 1 CAG repeat (t = 2,3; p<0,03; Fig. 3).

## Discussion

In a population of gifted and control boys 6 SNPs and 1 STR polymorphisms in genes related to testosterone metabolism were genotyped. The comparison of allele frequencies showed significant differences in the alleles of ESR2 and SHBG genes. Currently, these SNPs are not known to be functional and were chosen based on heterozygosity data from public databases. However, both genes play important roles in the brain metabolism of testosterone. It remains to be analyzed whether these SNPs have a direct molecular effect on testosterone transport, metabolism or recognition. SHBG concentrations and/or affinity to steroids determine the free bio-available fraction of testosterone [25]. This testosterone is in many brain areas further converted to estradiol and, thus, activates estrogen receptors [26]. Both genes, thus, encode important mediators of testosterone effects in all tissues, including the brain and might affect its morphology and physiology [27].

The functional polymorphism of the AR gene, widely studied in relation to androgen-dependent diseases, also seems to be associated with intellectual giftedness in boys. Although the difference in average number of CAG repeats is rather small, it is statistically significant and points towards a potential biological explanation of the relationship between testosterone and intelligence. Shorter alleles are known to have a higher DNA binding activity resulting in a stronger androgen signaling [28]. Hormonal levels were not assessed in this study, we, nevertheless, hypothesize that this stronger signaling during prenatal and early postnatal period results in lower testosterone levels later in the prepubertal period observed in our previous study [19].

It has been shown previously that the number of CAG repeats in exon 1 of the androgen gene is inversely associated with the transcriptional activity of the androgen receptor. The higher the number of CAG repeats, the lower the transcriptional activity of the androgen receptor that functions as transcription regulator as well [29]. This tandem repeat polymorphism was firstly described in the context of prostate cancer showing disequilibrium between particular tandem repeat genotypes in patients and healthy controls [30]. The polymorphic sequence can also be used to predict some urological problems, as lower number of alleles is associated with increased risk of prostate cancer in younger age, with increased morbidity and mortality [31], [32]. Although this polymorphism has been studied also with other pathological and physiological traits, our study is the first to look for and to find an association between CAG repeats in the androgen receptor gene and general intelligence.

The SNaPshot mini-sequencing technology is an interesting tool for analyzing several single nucleotide polymorphisms of candidate genes [33]. The DNA chip technology is largely used in studies without a priori hypothesis, which makes false positive results more probable. The candidate gene approach is more robust in this aspect, although the DNA chips are far more cost-effective. A major limitation of our study is a small sample size, but it is currently out of our possibilities to enlarge the sample in the near future. It cannot be ruled out that the associations found are specific for Slovak population of gifted boys, but it is unlikely due to genotype mixing in the central Europe.

In conclusion, our pilot study identifies three polymorphisms of genes related to testosterone metabolism in association with intellectual giftedness in boys. The higher trans-activational activity of the androgen receptor in gifted boys might explain the finding of lower testosterone levels in these children in a previous study [19]. It can be suggested that the genetic predisposition to increased androgen signaling leads to a more powerful negative feedback in the pituitary and lower needs of testosterone for the same androgen receptor-mediated effect. This low, but effective testosterone might, on the other hand, influence a number of androgen receptor independent mechanisms such as the non-genomic effects of steroids and the effect of testosterone mediated via estrogen receptors after aromatization to estradiol. In our previous study mentally challenged children had lower testosterone levels similarly to the intellectually gifted population. We did not include this group of patients into the current observation. High variability in the etiology of mental retardation will very likely prevent such findings in this group.

Although we have included nearly all available students of the school for gifted children, the sample size in this study is relatively small. The results must be taken with caution as this is a pilot study. Despite small number of participating children we have found significant differences in genotype frequencies. We, thus, think that the results should be published and be accessible for other research teams with better access to next-generation sequencing platforms and more DNA samples from intellectually gifted children. The control group has an average IQ far above 100 points, which might also decrease the sensitivity of the study. Replication of the found associations in larger multi-center study is, thus, needed. Testosterone measurements would be helpful in the interpretation of the associations. This was not possible in our study. To have at least this small sample size we included boys in puberty with high intraindividual variability of hormonal levels. Despite our selection of genes based on a rational choice from public databases, other genes related to testosterone metabolism might explain more variability of intelligence. These and other limitations should be overcome in further studies.
